# Assessment of the Genetic Diversity and Population Structure of a Wild Tea Germplasm Collection in Thailand Using SSR Markers

**DOI:** 10.3390/plants15162456

**Published:** 2026-08-13

**Authors:** Rungrote Nilthong, Phijittra Umalee, Somrudee Nilthong

**Affiliations:** 1 School of Science, Mae Fah Luang University, Chiang Rai 57100, Thailand; rungrote@mfu.ac.th (R.N.); 6151105005@lamduan.mfu.ac.th (P.U.); 2 Coffee Quality Research Center, Mae Fah Luang University, Chiang Rai 57100, Thailand; 3 Tea and Coffee Institute, Mae Fah Luang University, Chiang Rai 57100, Thailand

**Keywords:** *Camellia sinensis*, genetic diversity, population structure, SSR markers, tea plant

## Abstract

Tea [*Camellia sinensis* (L.) O. Kuntze] is the most widely consumed beverage worldwide and also has significant economic crop value. Understanding the genetic diversity and population structure of wild tea plants by analyzing their germplasm is essential for effective collection, management, and utilization. In this study, the genetic diversity and population structure of 283 wild tea accessions were analyzed using 16 SSR markers. A total of 81 alleles were detected, ranging from 3 to 8 alleles per marker, with an average of 5.06 alleles per locus. The average polymorphism information content (PIC) value of 0.545 indicates that the SSR marker set was, on average, highly informative. Moreover, the MSG0533 marker showed the presence of a distinct allele (350 bp) exclusively in tea accessions from the Chiang Mai province. This unique allele could be further developed into a DNA marker for accurate identification of tea sourced from this region. Based on the dendrogram, wild tea accessions were grouped into three major clusters exhibiting substantial genetic divergence. Population structure analysis showed two distinct subpopulations. Subpopulation 1 consists of 70 (24.73%) accessions from the provinces of Lampang, Mae Hong Son, Nan, and Phrae, whereas all 213 (75.27%) accessions in subpopulation 2 originate from the provinces of Chiang Mai and Chiang Rai. Analysis of molecular variance (AMOVA) identified 13% variance among and 56% variance within populations, while 31% was attributed to individuals, indicating a high gene exchange rate between the two subpopulations. These findings provide comprehensive information for future breeding and genetic studies of tea.

## 1. Introduction

Tea is one of the world’s oldest beverages and is the most popular nonalcoholic drink throughout the world, with an estimated 70% of the world’s population consuming it for its refreshing taste, appealing aroma, mild stimulating properties, and medicinal properties [1]. According to statistics from 2023 provided by the Food and Agriculture Organization of the United Nations [2], global total tea production (processed tea) has reached 6.6 million tons, a 1.9% rise from 2022. Tea is produced from the young shoot tips (two leaves and a bud) of tea plants (*Camellia sinensis* (L.) O. Kuntze, 2*n* = 2*x* = 30). In the thousands of years since the tea plant was first discovered, recorded, and cultivated for tea production in China, especially in the Yunnan and Sichuan regions and the northern part of Myanmar, tea has become widespread [3,4]. It is now cultivated in over 52 countries around the world, primarily in Asia, Africa, and South America [5,6]. Today, China and India are the two largest producers of tea globally and are expected to serve as a source of tea germplasm and planting materials for other countries [5,6,7]. The tea plant is a woody evergreen perennial plant with abundant secondary metabolites [8]. Tea plants exhibit self-incompatibility, are predominantly cross-pollinated, and can interbreed without any reproductive barriers. Consequently, the existing population has highly heterogeneous germplasm [9]. Based on morphological traits such as leaf size, flowers, and branching, tea plants are classified into two main taxa: *Camellia sinensis* var. *sinensis* (Chinese tea), characterized by small, dark green leaves and high cold tolerance; and *Camellia sinensis* var. *assamica* (Assam tea), distinguished by larger, lighter green leaves and adaptation to tropical climates [10]. Chinese tea exhibits the highest degree of self-incompatibility [11,12].

Genetic resources are essential sources for the selection and improvement of desirable traits through breeding [13,14]. However, the limited availability of genetic resources has slowed the advancement of tea breeding capacity. Interest in wild tea plants as new genetic resources for further tea improvement has increased. For plant breeding and germplasm management, it is important to collect, store, and evaluate germplasm resources [15,16]. Investigating genetic diversity and population structure is essential for understanding the natural selection history of and genetic relationships among tea plants. Evaluating genetic diversity through morphological traits is ambiguous, as these traits are influenced by environmental factors and are difficult to measure accurately [17,18]. In recent years, molecular markers have become more reliable because they can directly determine allelic diversity and provide accurate estimates of genetic distances. DNA molecular markers, including random amplified polymorphic DNA (RAPD) [19], amplified fragment length polymorphisms (AFLPs) [20], simple sequence repeats (SSRs) [21,22,23], expressed sequence tags (ESTs) [24], and inter-simple sequence repeats (ISSRs) [25,26] have shown potential for assessing genetic variation among tea varieties. Compared to other genetic markers, SSR has advantages including co-dominant inheritance, widespread genomic distribution, high transferability, high levels of polymorphism, and enhanced repeatability [27].

In Thailand, the Tea and Coffee Institute at Mae Fah Luang University has collected tea plants from highland forests, hillsides, and slopes, as well as higher areas with abundant water in the provinces of Chiang Mai, Chiang Rai, Nan, Phrae, Lampang, and Mae Hong Son in northern Thailand and has conserved these tea plants at the Singha Park tea germplasm bank, which is located in the Chiang Rai province of Thailand. To effectively manage, assess, and utilize the tea germplasm collection, it is essential to investigate the extent of genetic diversity that it contains.

The objectives of this study were to genotype 283 tea accessions using SSR markers to (1) evaluate genetic diversity, (2) investigate population structure, and (3) compare genetic properties among subpopulations.

## 2. Results

### 2.1. SSR Polymorphism

Sixteen SSR markers were used to determine genetic diversity among 283 wild tea accessions. A total of 81 alleles were detected using these primer pairs, with an average of 5.06 alleles per locus (Table 1). The number of observed alleles (*Na*) per locus varied from 3 alleles (MSE0143, MSG0681) to 8 alleles (MSG0533) (Table 1). The number of effective alleles (*Ne*) ranged from 1.711 (MSE0173) to 4.309 (MSE0083), with a mean value of 2.681 for all primer pairs. The MSG0533 marker has the highest number of polymorphic observed alleles (*Na*), but the number of effective alleles (*Ne*) was significantly lower (3.271). Six alleles per locus were observed for MSE0083, MSE0180, MSE0173, MSE0250, MSE0258, MSG0429, and D02T, showing a considerably wide range of effective alleles (*Ne*), from 1.711 (MSE0173) to 4.309 (MSE0083). The highest value (1.523) for the Shannon’s information index (*I*) was observed for the MSE0083 marker, while the lowest value (0.769) was found for the MSE0143 marker. The observed (*Ho*) and expected (*He*) heterozygosity values were highest for MSE0083. The minimum observed heterozygosity value was recorded for the MSE0143 marker, which exhibited the lowest level of polymorphism. This indicates that the MSE0143 marker has limited genetic variation, resulting in a lower likelihood of heterozygous individuals compared to the other markers analyzed [28]. The PIC value for each marker ranged from 0.728 (MSE0083) to 0.384 (MSE0173), with an average of 0.545. Furthermore, the MSG0533 marker showed a specific allele (350 bp) only in tea accessions originating in the Chiang Mai province. This specific allele could be further developed into a DNA marker for the precise identification and possible traceability and/or conservation of tea sourced from the Chiang Mai province.

### 2.2. Genetic Relationships and Population Structure

**Figure 1:**
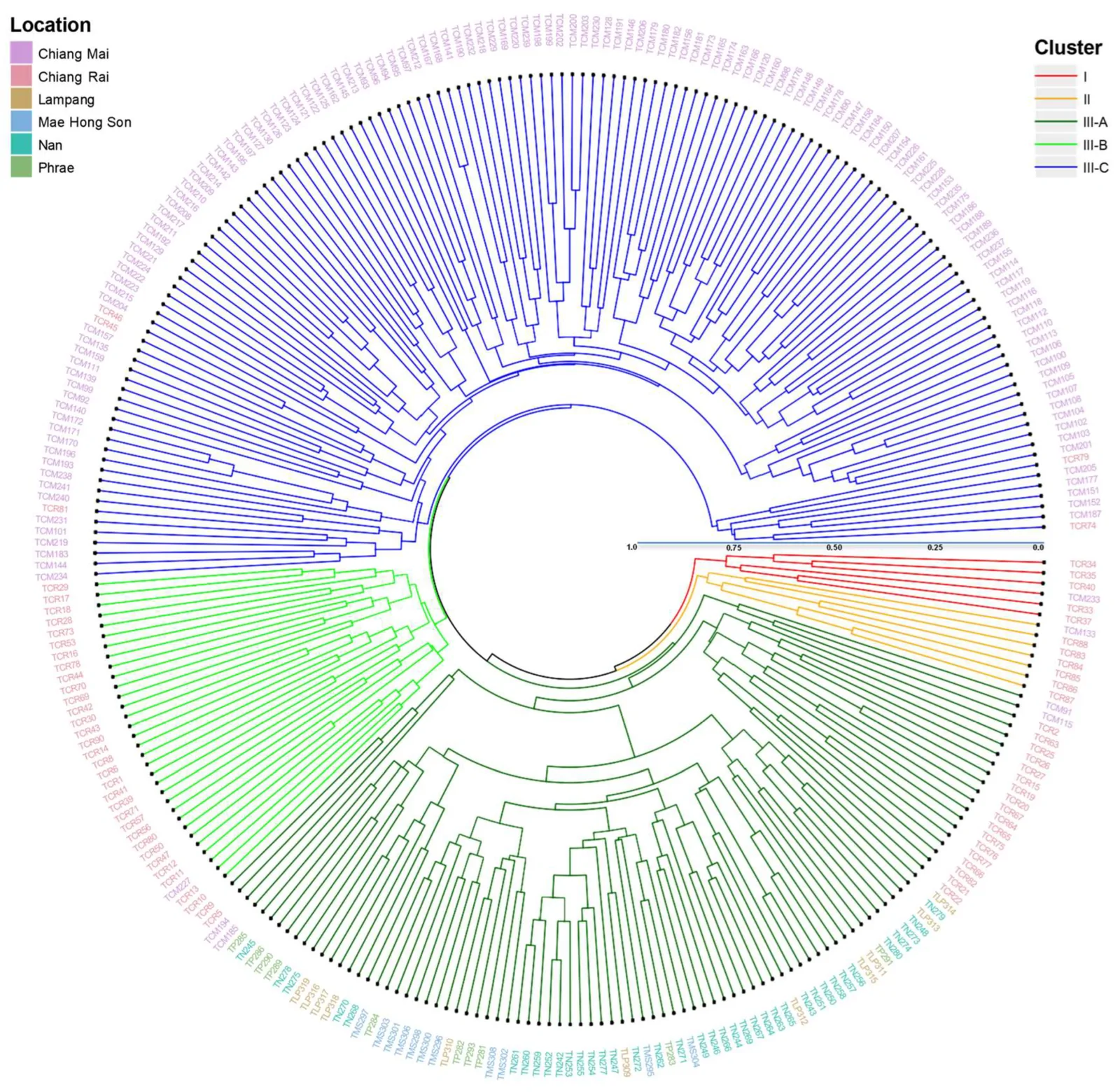
Unweighted pair group with arithmetic mean algorithm (UPGMA) dendrogram showing five groups of 283 wild tea accessions based on SSR data. The colors of the dendrogram lines indicate groupings, while the colors of the letters refer to the wild tea accessions.

SSR data were used to analyze the genetic relationships among 283 wild tea accessions using R program version 4.4.0 [29]. The genetic distance among accessions ranged from 0.188 to 0.756, demonstrating that the 283 wild tea accessions exhibited diverse genotypes. The unweighted pair group method with arithmetic mean (UPGMA)-based dendrogram classified the 283 wild tea accessions into three main clusters—Cluster I, Cluster II, and Cluster III—at a genetic distance of 0.847 (Figure 1). Cluster I consisted of six accessions (one from the Chiang Mai province and five from the Chiang Rai province), all of which were collected from Pang Rim Korn village in the Chiang Rai province. Cluster II comprised seven accessions (one from the Chiang Mai province and six from the Chiang Rai province). All five accessions from the Chiang Rai province originated from the same village (Pang Rim Korn village), although not all wild tea accessions from this village were included in this cluster. Cluster III, consisting of 270 accessions, was divided into three subclusters—III-A, III-B, and III-C at a genetic distance of 0.814. Subcluster III-A contained 94 accessions (Chiang Mai: 4; Chiang Rai: 20; Lampang: 11; Mae Hong Son: 11; Nan: 38; Phrae: 10). Subcluster III-B included 32 accessions (Chiang Mai: 1; Chiang Rai: 31). Subcluster III-C consisted of 144 accessions (Chiang Mai: 139; Chiang Rai: 5). All wild tea accessions that originated from the provinces of Lampang, Mae Hong Son, Nan, and Phrae were distributed within subcluster III-A. The majority of accessions from the Chiang Mai province were concentrated in subcluster III-C.

**Figure 2:**
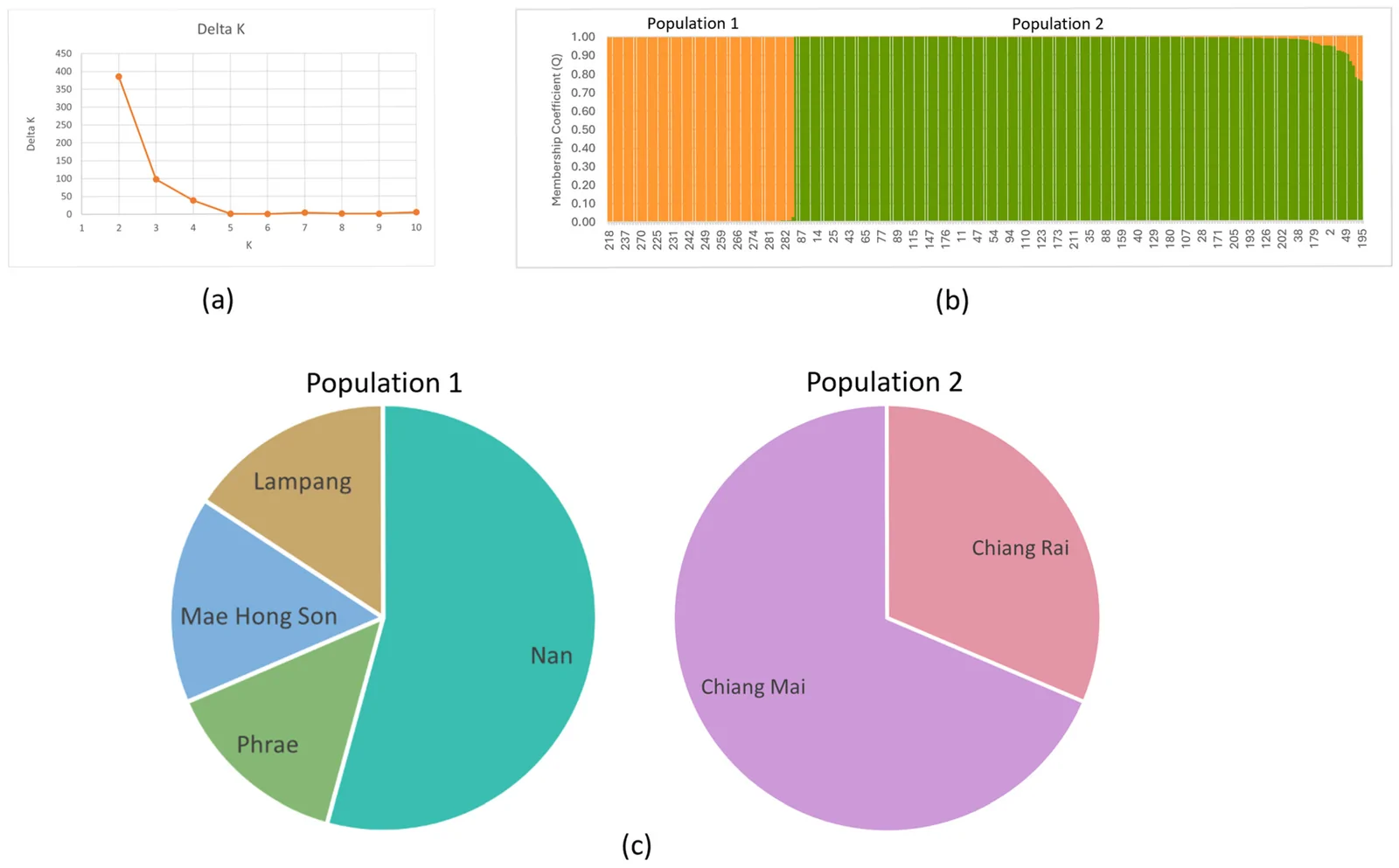
Population structure of the 283 wild tea accessions: (**a**) average log-likelihood of *K* value against *K*; (**b**) estimation of the population structure of the 283 wild tea accessions based on *K* = 2, where the *y*-axis corresponds to the subgroup membership, and the *x*-axis to the accession; (**c**) composition of location groups in the two subpopulations. Different location groups are represented by different colors.

STRUCTURE analysis software version 2.3.4 was used to study the population structure and determine the subpopulations (*K*) of the 283 wild tea accessions based on the distribution of the SSR alleles evaluated in this study. To select the optimum *K*-value for the best number of subpopulations, a line chart was drawn, as shown in Figure 2. It showed a clear maximum for the likelihood value (LnP(D)) derived delta *K* (Δ*K*) at *K* = 2 (Figure 2a). Therefore, a *K* value of 2 was chosen to demonstrate the genetic structure of the 283 wild tea accessions, indicating that all accessions could be split into two subpopulations (Figure 2b). Figure 2c shows the geographic compositions of the germplasm from the two subpopulations. The assignment of accessions into these inferred subpopulations was highly asymmetric. Subpopulation 1 (orange in Figure 2b) contained 70 (24.73%) accessions from different provinces—the Lampang, Mae Hong Son, Nan, and Phrae provinces—while all 213 (75.27%) accessions in subpopulation 2 (green in Figure 2b) were from the Chiang Mai and Chiang Rai provinces. The STRUCTURE results estimated the fixation index (*Fst*) for each of the subpopulations. Significant divergence was found within each of two subpopulations (Table 2). *Fst* values of 0.471 and 0.027 were obtained for subpopulation 1 and subpopulation 2, respectively (Table 2). Additionally, the average distances [expected heterozygosity (*He*)] between individuals within the same subpopulation were 0.6028 and 0.7954, respectively. The *He* of subpopulation 2 exceeded that of subpopulation 1, suggesting that subpopulation 2 exhibited more allelic diversity than subpopulation 1 (Table 2).

**Figure 3:**
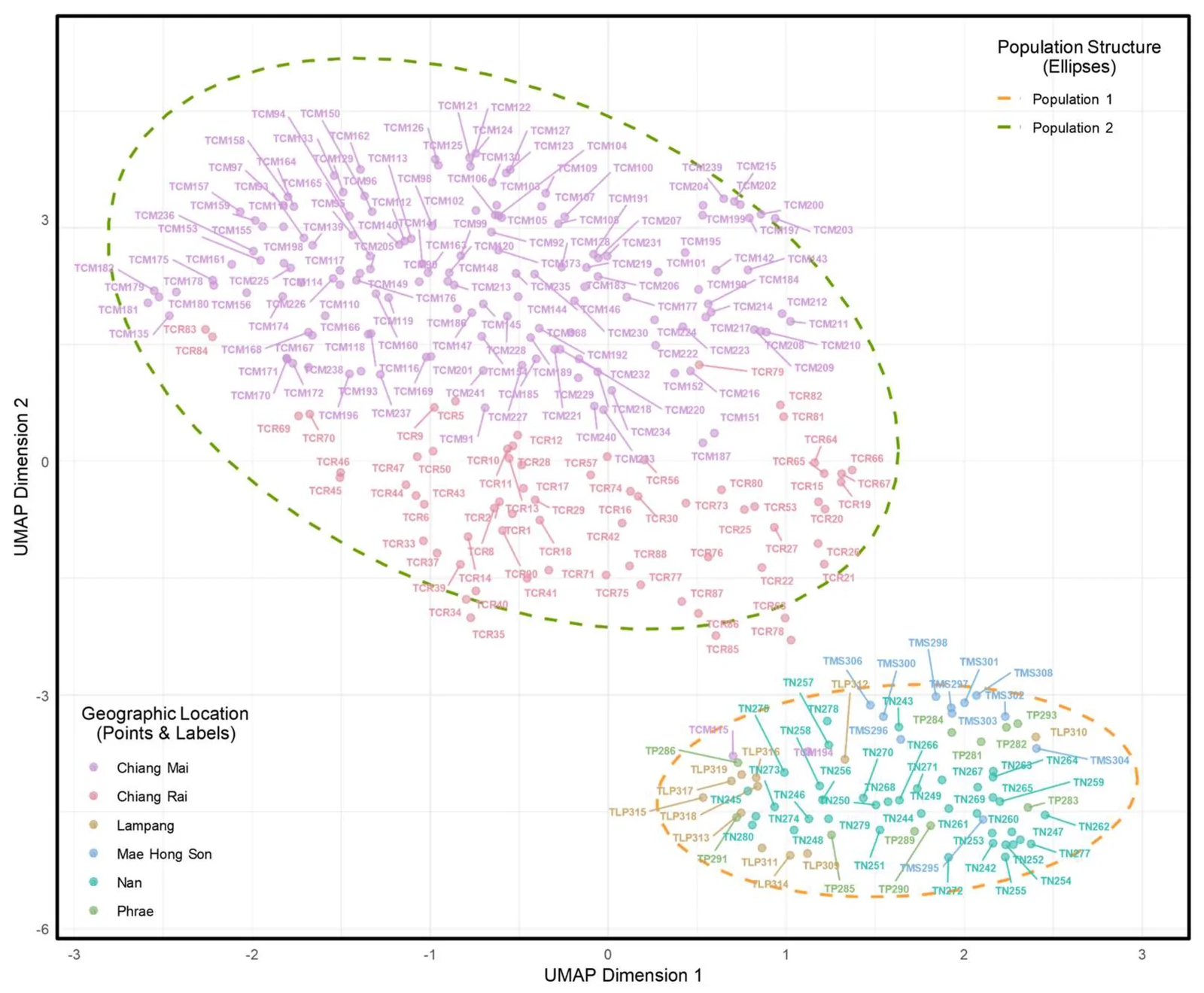
Distribution of the 283 wild tea accessions as determined using the UMAP (uniform manifold approximation and projection) method.

Uniform manifold approximation and projection (UMAP) analysis with SSR markers effectively divided the genetic structure of the 283 wild tea accessions into two major clusters along UMAP dimensions 1 and 2 (Figure 3) and showed a strong correlation with the genetic subdivisions found by the STRUCTURE analysis. The projection is clearly geographically organized. The tight and separated cluster in the lower right quadrant (surrounded by the population 1 circle; orange) distinctly differentiates accessions from the provinces of Lampang (TLP), Mae Hong Son (TMS), Nan (TN), and Phrae (TP), indicating significant local genetic affinity and obvious spatial structure among accessions from these geographic regions. In contrast, the large upper cluster, defined by the population 2 circle (green), was composed mainly of accessions from the Chiang Mai and Chiang Rai provinces and was characterized by the widely distributed TCM and TCR accessions.

### 2.3. Analysis of Molecular Variance

The percentage of genetic variation among populations, among individuals, and within populations was evaluated through analysis of molecular variance (AMOVA) using the obtained SSR marker data. The results from the AMOVA (Table 3) showed that most of the molecular variance occurred at the individual level. The highest molecular variation (56%) was distributed among wild tea varieties within populations, while 31% was attributed to individuals. Only a small proportion (13%) of variance was due to differences among populations. In addition, the pairwise fixation index (*Fst*) among the six wild tea populations ranged from 0.071 to 0.164 (Supplementary Table S1). The overall fixation index (*Fst*) among populations was 0.131 and showed strong significance (*p* < 0.001, based on 999 permutations). This indicates that, while six distinct genetic groups exist, the level of genetic differentiation among them is moderate, with high genetic diversity mostly found within each population. The estimated historical gene flow (*Nm*) among populations was 1.664 migrants per generation.

## 3. Discussion

Tea plants are cultivated in tropical and subtropical countries; a long history of cultivation and self-incompatibility make these plants highly heterogeneous, resulting in high diversity [30,31]. The evaluation of genetic diversity among the germplasm of various accessions promotes the efficient use of genetic resources.

### 3.1. Genetic Diversity

A total of 81 alleles were detected using SSR markers. The number of alleles per locus varied from 3 to 8, with an average of 5.06 alleles per locus. Bali et al. [9] similarly observed that the total number of alleles ranged from 2 to 14, with a mean of 6.67 alleles per locus. However, more alleles per locus (5–20) have been reported, with a mean value of 13.6, in Korean tea germplasm [22]. The variation between the allele number observed in this study and previously reported allele numbers may be attributed to the large population size and the degree of genetic variation within Korean tea populations [19].

The gene diversity of a locus, also referred to as expected heterozygosity (*He*), describes the proportion of heterozygosity expected under Hardy–Weinberg equilibrium. Both expected heterozygosity (*He*) and polymorphism information content (PIC) values are measures of genetic diversity among genotypes in breeding populations [32]. PIC values are a good indication of the usefulness of markers for linkage analysis in evaluating inheritance between offspring and parental genotypes [33], whereas expected heterozygosity (*He*) provides an estimate of the mean heterozygosity and genetic distance among individuals within a population [28,33]. In this study, the average values of *H_o_*, *H_e_*, and *I* derived from 16 SSR markers were 0.350, 0.601, and 1.122, respectively. These results show a similar level of genetic diversity to that previously reported by Taniguchi et al. [21] for *Camellia sinensis*. According to a previous study [34], markers with a PIC value of ≥0.5 were highly informative. If the PIC value ranged from 0.25 to 0.50, the markers were moderately informative, but markers with a PIC value below 0.25 were only slightly informative. Our results showed that the PIC values for all the markers were 0.545, indicating that the SSR marker set was, on average, highly informative, although individual markers ranged from moderately to highly informative. The present finding is similar to those previously published by Bali et al. [9], who assessed 21 tea clones using 22 SSR markers and reported a mean PIC value of 0.6.

### 3.2. Genetic Relationships and Population Structure

The UPGMA-based dendrogram classified the 283 wild tea accessions into three main clusters at a genetic distance of 0.847, as shown in Figure 1. Cluster I comprised six accessions, while cluster II included seven accessions. Cluster III was the largest cluster, comprised of 270 accessions, and was further subdivided into three subclusters. Population structure analysis is informative for understanding genetic diversity [35]. Bayesian clustering analysis showed a highly asymmetric population structure in terms of genetic differentiation (*Fst*) and distribution of accessions among the inferred subpopulations. The results showed a core and satellite demographic model, whereas a large, continuous genetic reservoir is associated with a significantly smaller and highly isolated splinter population. STRUCTURE-derived fixation index (*Fst*) values measure the genetic divergence of each inferred population relative to the inferred ancestral population, rather than overall genetic differentiation between populations. Populations were considered to have very strong differentiation when *Fst* values were greater than 0.25, strong differentiation when *Fst* values were between 0.15 and 0.25, moderate differentiation when *Fst* values were between 0.05 and 0.15, and little differentiation when *Fst* values were less than 0.05 [36,37]. As seen in Table 2, subpopulation 1 comprises a small percentage (24.8%) of the gene pool, but this subpopulation is highly genetically differentiated (*Fst* = 0.471) from the ancestral gene pool, suggesting severe isolation. Because this subpopulation is a small percentage of the whole data set and is more sensitive to genetic drift, the significant divergence seen is probably due to a population bottleneck, founder effects, or prolonged geographical and reproductive isolation that limited gene flow with the core subpopulation [38]. A dominant genetic cluster (75.3%) was observed in subpopulation 2. The low fixation index (*Fst* = 0.027) of subpopulation 2 indicates small divergence from the ancestral gene pool, according to Wright [36], suggesting that this subpopulation is still genetically close to the inferred ancestral population. From a biological perspective, the low *Fst* value and high percentage of accessions indicate that subpopulation 2 is undergoing significant, continuous gene flow. This group is probably the major, enduring genetic source of wild tea in the studied area, where a large effective population size prevents the rapid fixation of alleles by genetic drift [39]. However, unbalanced accession numbers among provinces can affect the detection of rare alleles and population structure in provinces with large accession numbers. STRUCTURE may also be sensitive to unbalanced accession numbers and may struggle to detect small genetic clusters [40].

UPGMA is a distance-based, hierarchical agglomerative method that uses pairwise genetic distances. This method organizes accessions into a tightly branched structure, which means a lack of ability to continue gene flow, hybridization, or admixture. Individuals with intermediate genetic profiles are often grouped into their own distinct branches, leading to an increase in the number of clusters. Although Bayesian STRUCTURE analysis is a model-based probabilistic algorithm that classifies individuals into *K* (*K* = 2) populations by reducing differences from Hardy–Weinberg equilibrium and linkage disequilibrium [41], the admixture model in STRUCTURE allows individuals to share fractional membership across several subpopulations, resulting in a more accurate depiction of complex evolutionary histories, including gene flow. The results showed that UPGMA cluster III-A was embedded in STRUCTURE subpopulation 1, which was a highly isolated and genetically distinct lineage (*Fst* = 0.471). UPGMA clusters I, II, III-B, and III-C were found in STRUCTURE subpopulation 2. UPGMA divided subpopulation 2 into several branches, possibly indicating small regional differentiation, minor allele frequency variations, or isolation by distance within the core gene pool. However, due to the low differentiation (*Fst* = 0.027) within subpopulation 2, these distance-based differences were not sufficient to violate Hardy–Weinberg expectations and justify separate subpopulation classification. Therefore, the UPGMA and STRUCTURE results are not contradictory. STRUCTURE identifies the two significant ancestral lineages that shape the wild tea populations, whereas UPGMA shows the more intricate, distance-based regional substructure within that large core population.

The level of genetic divergence among the designated geographic populations was evaluated using AMOVA and pairwise *Fst* analyses. The AMOVA results (Table 3) showed that 13% of the genetic variance occurred among subpopulations, resulting in an *Fst* value of 0.131. Based on Wright’s qualitative guidelines [36], an *Fst* value between 0.05 and 0.15 indicates moderate genetic differentiation. This suggests that the populations are not completely isolated, but that geographic, environmental, or historical barriers have caused significant genetic differences. The majority of genetic diversity (56%) was found within subpopulations, while 31% of total variation was due to differences among individuals within the population. These results are consistent with previous research by Lee et al. [22], which identified a high genetic diversity of 99% within populations and a very low genetic diversity of 1% within populations among 410 tea accessions from the Korea Genebank. The low molecular variance among subpopulations (13%) suggests limited differentiation of populations, indicating that tea plants from the six provinces have not been exposed to strong selection or genetic drift [21]. The estimated rate of gene flow (*Nm*) directly supports this moderate genetic differentiation in the population. Wright [42] reported that gene flow (*Nm*) helps increase the genetic diversity in plant populations and has a strong effect on genetic differentiation. An *Nm* value of less than 1 means limited gene exchange among subpopulations. In this study, the gene flow (*Nm*) value among subpopulations was 1.664, indicating high genetic exchange or high gene flow, which resulted in low genetic differentiation between subpopulations [35].

Furthermore, the results of uniform manifold approximation and projection (UMAP) analysis based on SSR markers were largely in agreement with those obtained from the population structure analysis. Population 1 was composed of accessions from the provinces of Lampang (TLP), Mae Hong Son (TMS), Nan (TN), and Phrae (TP), whereas population 2 was composed mainly of accessions from the provinces of Chiang Mai (CM) and Chiang Rai (CR). Accessions in population 2 exhibited a broader dispersion across local neighborhoods compared to the more compact clustering of population 1. In several tests with different random seed evaluations, these local neighborhood clusters showed topological stability, confirming that the spatial arrangement reflects consistent genetic similarities among accessions. These intricate local clustering patterns provide essential foundational information for germplasm classification and parental selection in future breeding programs.

## 4. Materials and Methods

### 4.1. Tea Materials and Genomic DNA Extraction

**Figure 4:**
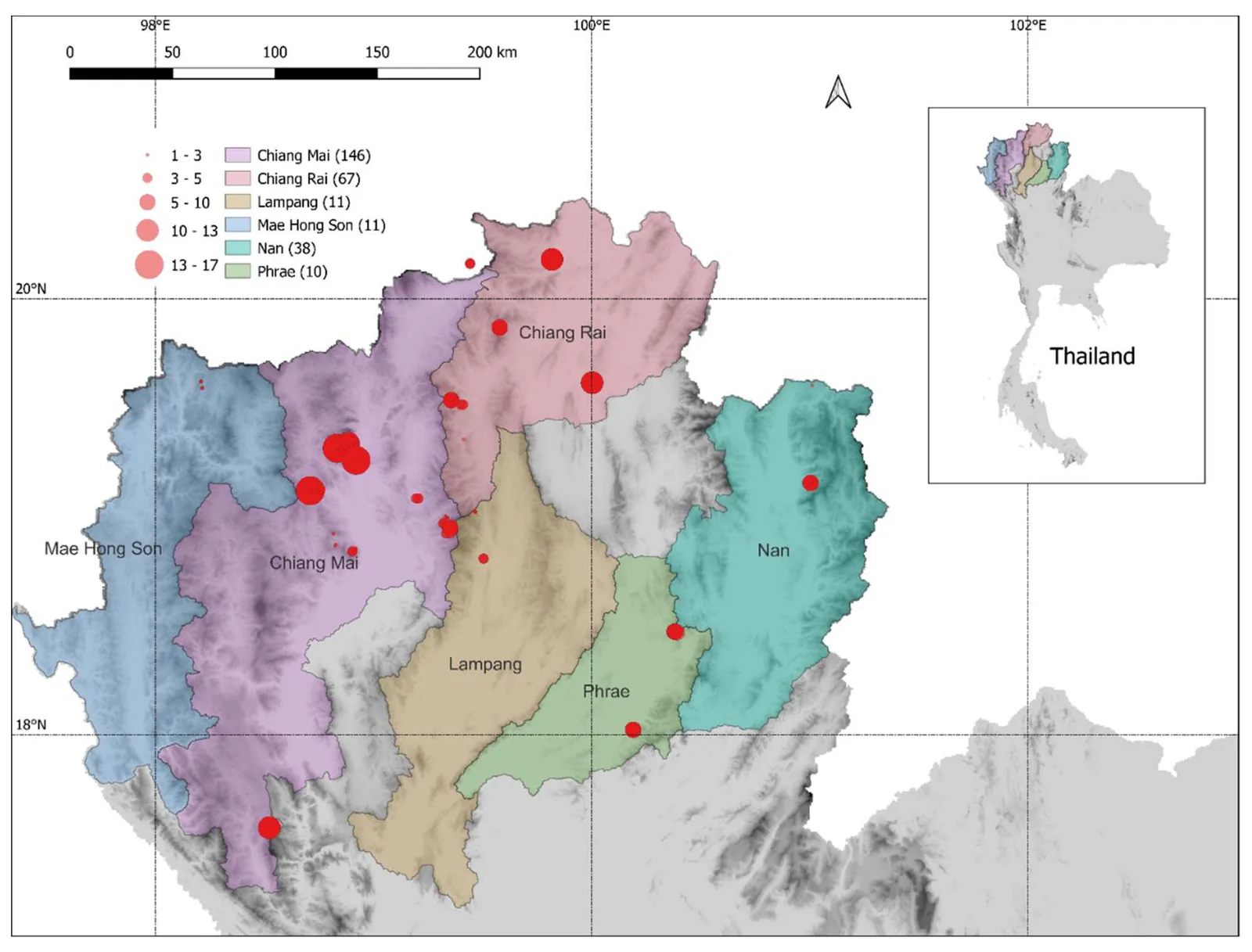
Map of the geographical distribution of the 283 wild tea accessions used in this study.

A total of 283 wild tea accessions obtained from the Singha Park tea germplasm bank, Chiang Rai province, Thailand, were used in this study (Supplementary Table S2). Among 283 wild tea accessions, 146 were initially gathered from highland forests, hillsides, and slopes, as well as higher areas with abundant water in the Chiang Mai province; 67 from the Chiang Rai province; 37 from the Nan province; 11 from the Phrae province; 11 from the Lampang province; and 11 from the Mae Hong Son province (Figure 4). Genomic DNA was extracted from young leaves using a TIANGEN DNA extraction kit (TIANGEN Biotech (Beijing) Co., Ltd., Beijing, China) according to the manufacturer’s guidelines. The extracted DNA was quantified using a Nanodrop spectrophotometer (Thermo Fisher Scientific, Waltham, MA, USA), and its quality was evaluated by 3% MetaPhor^®^ (Lonza, Rockland, ME, USA) agarose gel electrophoresis. The DNA was diluted with 1× TE buffer to a concentration of 50 ng/µL and stored at −20 °C for future use.

### 4.2. SSR Marker Analysis

Twenty-six SSR markers were selected from previously reported SSR markers [21,43] to screen for polymorphisms in the 283 wild tea accessions. These markers were selected based on previous work and successful application to a global core collection of *Camellia sinensis* [21,43]. These markers demonstrated high reproducibility, clear allelic scoring, and high polymorphism information content (PIC). The 16 SSR markers (Table 1) were used in the SSR analysis. The remaining markers were excluded because of poor amplification. The PCR reaction was performed in a 20 μL reaction mixture, containing 100 ng of genomic DNA, 1.25 mM of MgCl_2_, 1× PCR buffer, 200 μM of dNTPs, 5 pmol of each primer (Bionics, Seoul, Republic of Korea), and 1 U of *Taq* Polymerase (Vivantis, Selangor, Malaysia). The PCR reactions were carried out using an Eppendorf Mastercycler Nexus Gradient GSX1 Thermal Cycler (Eppendorf, Hamburg, Germany) according to the following “touchdown PCR” cycling program: 95 °C for 5 min, 95 °C for 1 min, 60 °C for 30 s, and 72 °C for 1 min; 10 cycles at annealing temperatures decreasing by 1 °C per cycle; 30 cycles of 95 °C for 1 min, 45 °C for 2 min, and 72 °C for 1 min; and a final extension step at 72 °C for 5 min. PCR products were separated by 3% agarose gel electrophoresis in 1× TAE buffer at 100 V for 1.5 h. The gels were visualized under UV light using a gel documentation system (Bio-Rad, Feldkirchen, Germany). Allele sizes were estimated using a 50 bp DNA Ladder (SMOBIO Technology Inc., Frederick, MD, USA).

### 4.3. Analysis of Genetic Diversity and Population Structure

The amplified fragment sizes of the different accessions at each SSR locus were used to calculate the number of alleles and allele frequencies. Polymorphic information content (PIC), a measure of the allelic diversity at a locus, was calculated in accordance with Anderson et al. [44] utilizing the equation $PIC_{i}=1-\sum_{j}{f_{ij}}^{2}$, where $f_{ij}$ is the frequency of the *j*th pattern (present and absent) of the *i*th band. Next, the PIC of each primer was calculated as $PIC=(\sum_{i=1}^{n}{PIC}_{i})/n$, where *n* is the number of bands. An unweighted pair group method with the arithmetic mean (UPGMA) dendrogram was constructed using the R program version 4.40 [28] based on the coancestry coefficient obtained from the pairwise shared allele distance matrices.

A Bayesian model-based clustering analysis was performed using STRUCTURE version 2.3.4 [45] to examine the genetic relationship among the wild tea germplasm samples using co-dominant genotypic data. The subpopulation number (*K*) was estimated using varying *K*-values from *K* = 1 to *K* = 10, with the analysis conducted ten times for each value. The burn-in time and the number of Monte Carlo Markov Chain replications were both established at 100,000 for each iteration. Then, the peak value of Δ*K*, derived from the variation in the log probability of the data [LnP(D)] between successive *K*-values, was used to determine the optimal *K*-value, representing the number of genetically distinct clusters inside the data [45]. Analysis of molecular variance (AMOVA) was performed using GenAlEX 6.503 software [41], with 999 permutations. The fixation index (*Fst*), number of different alleles (*Na*), Shannon information index (*I*), gene flow (*Nm*), expected heterozygosity (*He*), and observed heterozygosity (*Ho*) of each locus were calculated using GenAlEX 6.503 software [46]. Gene flow (*Nm*) among subpopulations was estimated from pairwise genetic differentiation using the equation $N_{m}=\frac{1-F_{ST}}{4F_{ST}}$, where *Fst* is the pairwise fixation index (Supplementary Table S1). Pairwise *Nm* values were calculated for all combinations of subpopulations, and the average gene flow was obtained as the arithmetic mean of these pairwise estimates [36].

The uniform manifold approximation and projection (UMAP) [47] method was implemented using the UMAP package (v0.2.10.0) in R (v4.4.0) [28] to generate nonlinear dimensionality reduction and visualization based on the shared allele distance matrix. This approach was used to evaluate fine-scale local neighborhood relationships among accessions. To ensure exact reproducibility, UMAP parameters were fixed at n_neighbors = 15, min_dist = 0.1, metric = “euclidean”, and random_state = 789. Multiple random seeds were evaluated to confirm that cluster membership and local neighborhood topology remained consistent across runs regardless of initial stochastic orientation.

## 5. Conclusions

This study analyzed 283 wild tea accessions using 16 SSR markers to investigate genetic diversity and population structure based on tea germplasm. Our findings indicate that the genetic diversity among the 283 wild tea accessions was diverse. Based on the dendrogram, the 283 wild tea accessions were clustered into three main clusters. Additionally, the finding of a unique MSG0533 allele was a significant advancement for the accurate identification of tea originating from the Chiang Mai province. Further studies of the biochemical components of tea accessions are essential to understanding the characteristics of tea accessions and promoting utilization of tea germplasm. Our results have important implications for the conservation, management, and utilization of tea germplasm.

## Figures and Tables

**Table 1:** List of markers used, sequences, number of alleles (*Na*), number of effective alleles (*Ne*), observed heterozygosity (*Ho*), expected heterozygosity (*He*), Shannon’s information index (*I*), and polymorphic information content (PIC) value.

SSR Primers	Primer Sequences (5′–3′)	*Na*	*Ne*	*Ho*	*He*	*I*	PIC
MSE0083	F: GAG GAA AGA GAT TAT GCG GTG TGG R: GTG AGC CTT CAA AAG ACA GCA ACG	6	4.309	0.940	0.768	1.523	0.728
MSE0107	F: TCT CTC TAC TCC TGC GCA ATC TCA R: TCA AAG ATG TTG CTC TCG TCA ACC	4	1.770	0.230	0.435	0.857	0.405
MSE0108	F: AGT CCA TGG TGG TTG ATG ATC CTT R: TTG GGA GTA GGA TTC TTG CAG AGC	6	3.465	0.424	0.711	1.391	0.664
MSE0143	F: GCC TTT TTG TCA GAA ACG GTG ACT R: CAG CAA TCT TTG GTT TTG TTG TGC	3	2.069	0.011	0.517	0.769	0.400
MSE0173	F: GTG TTC ACC AAC AAC TCA CCA AGG R: TGT CGA ACA AAG ATA CAC CCC AAA	6	1.711	0.141	0.416	0.808	0.384
MSE0237	F: CTC TCC TTC TTC ACA CCC TCC AAA R: TTG TTC TCA AAG AAC CTC CTT CGC	4	2.429	0.265	0.588	1.063	0.532
MSE0250	F: CTT CCC CAA ACC ACC ATC AAA ATA R: GAA ATT GAA GAA CAC GAA CCT GCC	6	2.685	0.357	0.628	1.198	0.576
MSG0258	F: ACT CAT CAC CAT GCC TTC TCC AT R: GTT TAG CTC AAC TGG TGG AAC CTC AAC T	6	3.067	0.728	0.674	1.317	0.628
MSE0313	F: TGC TAT GCC GCC TAA CAA AAA CTT R: ACC ACC AAC AAC AAT TCC CAC TCT	4	2.425	0.106	0.588	1.068	0.532
MSG0403	F: ATG ATC GCC GGT TTA GAG ATG AAT R: GTT TAA GCT GGC TAA CCT ACA CGG AGC	5	2.109	0.339	0.526	0.870	0.429
MSG0429	F: AGG ACC GTT CTT CCC TAC CTG TAA R: GTT TGA GAT TGA GGA TGT GGT CGT TGT	6	2.628	0.413	0.619	1.200	0.569
MSG0470	F: ATA GGG TTC GAA AAT GGC AGG R: GTT TGA GGT GGC AAG TTT GTG ACT GT	4	3.819	0.565	0.738	1.363	0.690
MSG0533	F: AGA CCT AGC CAA GAC AAC CAC ACC R: GTT TCC CCT ATT TTC CCG ACT GTC T	8	3.271	0.067	0.694	1.401	0.644
MSG0610	F: ACA GAG GAG GAA GAT GAT CGG TAA R: GTT TGA AGA AGA AGA AAA CTC CCG CCA T	4	2.681	0.428	0.627	1.110	0.555
MSG0681	F: AGG GTT TGC GTC TTC AAA GAG AGA R: GTT TGT AAC ACT TGC CAC GTT TCG	3	1.929	0.251	0.481	0.817	0.420
D02T	F: ATG TAG CAT TTT GGC TTA TTG GAT CAC R: GTT TCC TGT CAA AGC TCC TCT TGT GG	6	2.523	0.339	0.604	1.192	0.565
	Mean	5.063	2.681	0.350	0.601	1.122	0.545
	St. Dev	1.439	0.742	0.243	0.104	0.242	0.028

Note: *Na*, number of alleles; *Ne*, number of effective alleles; *Ho*, observed heterozygosity; *He*, expected heterozygosity; *I*, Shannon’s information index; PIC, polymorphic information content.

**Table 2:** STRUCTURE results from 283 wild tea accessions for the fixation index (*Fst*), average distances (expected heterozygosity/*He*), and number of genotypes assigned to each subpopulation.

Subpopulation	Inferred Clusters	Mean *Fst*	Expected Heterozygosity (*He*)	No. of Genotypes
Subpopulation 1	0.248	0.471	0.603	70
Subpopulation 2	0.753	0.027	0.795	213

**Table 3:** Analysis of molecular variance (AMOVA) between, among, and within subpopulations of the 283 wild tea accessions.

Source	*df*	SS	MS	Est. Var.	Variance (%)
Among populations	5	274.344	54.869	0.659	13
Among individuals	277	1653.317	5.969	1.583	31
Within populations	283	793.000	2.802	2.802	56
Total	565	2720.661		5.044	100
Fixation index (*Fst*)	0.131				
*Nm*	1.664				

Note: *df* indicates degrees of freedom; SS, sums of squares; MS, mean squares; Est. Variance, estimated variance component; Nm, gene flow.

## Data Availability

The original contributions presented in this study are included in the article/Supplementary Material. Further inquiries can be directed to the corresponding author.

## Supplementary Materials

The following supporting information can be downloaded at: https://www.mdpi.com/article/10.3390/plants15162456/s1, Table S1. Pairwise Population *Fst* Values. Table S2. List of wild tea plants used in this study. Name, code, origin, latitude and longitude of provinces in Thailand.

## References

[1] Wang C., Han J., Pu Y., Wang X. (2022). Tea (*Camellia sinensis*): A review of nutritional composition, potential applications, and omics research. Appl. Sci..

[2] Food and Agriculture Organization of the United Nations. https://openknowledge.fao.org/server/api/core/bitstreams/e1d8588a-ddba-4b49-9897-311611391a76/content.

[3] Zhang X., Chen S., Shi L., Gong D., Zhang S., Zhao Q., Zhan D., Vasseur L., Wang Y., Yu J. (2021). Haplotype-resolved genome assembly provides insights into evolutionary history of the tea plant *Camellia sinensis*. Nat. Genet..

[4] Yu F.L. (1986). Discussion on the originating place and the originating center of tea plants. J. Tea Sci..

[5] Kong W., Kong X., Xia Z., Li X., Wang F., Shan R., Chen Z., You X., Zhao Y., Hu Y. (2025). Genomic analysis of 1,325 Camellia accessions shed light on agronomic and metabolic traits for tea plant improvement. Nat. Genet..

[6] Mjanja B.A., Kang Y., Galal A. (2025). A systematic review on criteria for land suitability assessment for tea cultivation. Front. Agron..

[7] Wambulwa M.C., Meegahakumbura M.K., Kamunya S., Muchugi A., Möller M., Liu J., Xu J.C., Li D.Z., Gao L.M. (2017). Multiple origins and a narrow genepool characterise the African tea germplasm: Concordant patterns revealed by nuclear and plastid DNA markers. Sci. Rep..

[8] Wight W. (1959). Nomenclature and classification of the tea plant. Nature.

[9] Bali S., Raina S.N., Bhat V., Aggarwal R.K., Goel S. (2013). Development of a set of genomic microsatellite markers in tea (*Camellia* L.) (*Camellianceae*). Mol. Breed..

[10] Ming T.L., Bartholomew B., Wu Z.Y., Raven P.H., Hong D.Y. (2007). Theaceae. Flora of China.

[11] Wight W. (1956). Commercial selection and breeding of tea in India. World Crops.

[12] Sealy J.R. (1958). A Revision of the Genus Camellia.

[13] Fu Y.R., Liu F.L., Li S., Tian D.K., Dong L., Chen Y.C., Su Y. (2021). Genetic diversity of the wild Asian lotus (*Nelumbo nucifera*) from northern China. Hortic. Plant J..

[14] Guo Q., Liu J., Li J., Cao S., Zhang Z., Zhang J., Zhang Y., Deng Y., Niu D., Su L. (2022). Genetic diversity and core collection extraction of *Robinia pseudoacacia* L. germplasm resources based on phenotype, physiology, and genotyping markers. Ind. Crop Prod..

[15] Boczkowska M., Łapiński B., Kordulasińska I., Dostatny D.F., Czembor J.H. (2016). Promoting the use of common oat genetic resources through diversity analysis and core collection construction. PLoS ONE.

[16] Cuevas H.E., Prom L.K. (2020). Evaluation of genetic diversity, agronomic traits, and anthracnose resistance in the NPGS Sudan Sorghum Core collection. BMC Genom..

[17] Chen R.K., Hara T., Ohsawa Y. (2017). Analysis of genetic diversity of rapeseed genetic resources in Japan and core collection construction. Breed. Sci..

[18] Ouni R., Zborowska A., Sehic J., Choulak S., Hormaza J.I., Garkava-Gustavsson L., Mars M. (2020). Genetic diversity and structure of Tunisian local pear germplasm as revealed by SSR markers. Hortic. Plant J..

[19] Kaundun S.S., Zhyvoloup A., Park Y.G. (2000). Evaluation of the genetic diversity among elite tea (*Camellia sinensis* var. sinensis) accessions using RAPD markers. Euphytica.

[20] Kaundun S.S., Matsumoto S. (2002). Heterologous nuclear and chloroplast microsatellite amplification and variation in tea *Camellia sinensis*. Genome.

[21] Taniguchi F., Kimura K., Saba T., Ogino A., Yamaguchi S., Tanaka J. (2014). Worldwide core collections of tea (*Camellia sinensis*) based on SSR markers. Tree Genet. Genomes.

[22] Lee K.J., Lee J.R., Sebastin R., Shin M.J., Kim S.H., Cho G.T., Hyun D.Y., Lee K.J. (2019). Assessment of genetic diversity of tea germplasm for Its management and sustainable use in Korea genebank. Forests.

[23] Li M.M., Meegahakumbura M.K., Wambulwa M.C., Burgess K.S., Möller M., Shen Z.F., Li D.Z., Gao L.M. (2024). Genetic analyses of ancient tea trees provide insights into the breeding history and dissemination of Chinese Assam tea (*Camellia sinensis* var. assamica). Plant Divers..

[24] Zhang Y., Zhang X., Chen X., Sun W., Li J. (2018). Genetic diversity and structure of tea plant in Qinba area in China by three types of molecular markers. Hereditas.

[25] Montahae D.S., Rezaei M.B., Ghanbari J.M., Kalateh J.S., Jahangirzadeh K.S. (2023). Genetic resources diversity of tea (*Camellia sinensis* (L.) Kuntze) in the southern region of the Caspian Sea. Plant Genet. Resour..

[26] Yao M., Chen L., Liang Y. (2008). Genetic diversity among tea cultivars from China, Japan, and Kenya revealed by ISSR markers and its implication for parental selection in tea breeding programmes. Plant Breed..

[27] Park Y.J., Lee J.K., Kim N.S. (2009). Simple sequence repeat polymorphisms (SSRPs) for evaluation of molecular diversity and germplasm classification of minor crops. Molecules.

[28] Team R.C. (2015). R a Language and Environment for Statistical Computing. http://www.r-project.org.

[29] Chen L., Gao Q.K., Chen D.M., Xu C.J. (2005). The use of RAPD markers for detecting genetic diversity, relationship and molecular identification of Chinese elite tea genetic resources [*Camellia sinensis* (L.) O. Kuntze] preserved in a tea germplasm repository. Biodivers. Conserv..

[30] Kottawa-Arachchi J.D., Gunasekare M.T.K., Ranatunga M.A.B. (2019). Biochemical diversity of global tea [*Camellia sinensis* (L.) O. Kuntze] germplasm and its exploitation: A review. Genet. Resour. Crop Evol..

[31] Harris A.M., DeGiorgio M. (2017). An unbiased estimator of gene diversity with improved variance for samples containing related and inbred individuals of any ploidy. G3.

[32] Shete S., Tiwari H., Elsto R.C. (2000). On estimating the heterozygosity and polymorphism information content value. Theor. Popul. Biol..

[33] Nei M. (1990). Heterozygosity and genetic distance a citation classic commentary on estimation of average heterozygosity and genetic distance from a small number of individuals. Genetics.

[34] Botstein D., White R.L., Skolinick M., Davis R.W. (1980). Construction of a genetic linkage map in man using restriction fragment length polymorphisms. Am. J. Hum. Genet..

[35] Eltaher S., Sallam A., Belamkar V., Emara H.A., Nower A.A., Salem K.F.M., Poland J., Baenziger P.S. (2018). Genetic diversity and population structure of F3:6 Nebraska winter wheat genotypes using genotyping-by-sequencing. Front. Genet..

[36] Wright S. (1978). Evolution and the Genetics of Populations, Volume 4: Variability Within and Among Natural Populations.

[37] Mohammadi S.A., Prasanna B.M. (2003). Analysis of genetic diversity in crop plants—Salient statistical tools and considerations. Crop Sci..

[38] Frankham R., Ballou J.D., Briscoe D.A. (2010). Introduction to Conservation Genetics.

[39] Slatkin M. (1987). Gene flow and the geographic structure of natural populations. Science.

[40] Puechmaille S.J. (2016). The program STRUCTURE does not reliably recover the correct population structure when sampling is uneven: Subsampling and new estimators alleviate the problem. Mol. Ecol. Resour..

[41] Pritchard J.K., Stephens M., Donnelly P. (2000). Inference of population structure using multilocus genotype data. Genetics.

[42] Wright S. (1965). The interpretation of population structure by F-statistics with special regard to systems of mating. Evolution.

[43] Kato F., Taniguchi F., Monobe M., Ema K., Hirono H., Maeda-Yamamoto M. (2008). Identification of Japanese tea (*Camellia sinenesis*) cultivars using SSR marker. J. Jpn. Soc. Food Sci..

[44] Anderson J., Churchill G., Autrique J., Tanksley S., Sorrells M. (1993). Optimizing parental selection for genetic linkage maps. Genome.

[45] Evanno G., Regnaut S., Goudet J. (2005). Detecting the number of clusters of individuals using the software STRUCTURE: A simulation study. Mol. Ecol..

[46] Peakall R., Smouse P. (2012). GenAlEx 6.5: Genetic analysis in Excel. Population genetic software for teaching and research-an update. Bioinformatics.

[47] McInness L., Healy J., Saul N., Großberger L. (2018). UMAP: Uniform manifold approximation and projection. J. Open Source Softw..

