# Supplementary material for: Assessment of the Genetic Diversity and Population Structure of a Wild Tea Germplasm Collection in Thailand Using SSR Markers
Source: Plants (Basel). 2026 Aug 13;15(16):2456. doi: 10.3390/plants15162456 (PMC13516499; doi:10.3390/plants15162456)
Supplement: Supplementary file 1 [file plants-15-02456-s001.zip › plants-4443938-supplementary.pdf]

Table S1 Pairwise Population *Fst* Values.

	CR	CM	Nan	Prae	MS	LP
**CR**						
**CM**	0.113					
**Nan**	0.142	0.152				
**Prae**	0.149	0.155	0.031			
**MS**	0.133	0.164	0.088	0.071		
**LP**	0.096	0.150	0.073	0.088	0.109	

Note: CR, Chiang Rai province; CM, Chaing Mai province; MS, Mae Hong Son province; LP, Lampang province.

Table S2 List of wild tea plants used in this study. Name, code, origin, latitude and longitude of provinces in Thailand

Code	Geographical origin	Latitude and longitude	Alt. (m)	Cl.	Code	Geographical origin	Latitude and longitude	Alt. (m)	Cl.
TCR1	Chiang Khian, CR	N 19.3652 E 99.59532	398	IIIB	TCR2	Chiang Khian, CR	N 19.3652 E 99.59532	398	IIIA
TCR5	Chiang Khian, CR	N 19.3652 E 99.59532	398	IIIA	TCR6	Chiang Khian, CR	N 19.3656 E 99.59534	402	IIIB
TCR8	Chiang Khian, CR	N 19.3652 E 100.00236	425	IIIB	TCR9	Chiang Khian, CR	N 19.3652 E 100.00236	425	IIIA
TCR10	Chiang Khian, CR	N 19.3652 E 100.00236	425	IIIB	TCR11	Chiang Khian, CR	N 19.3652 E 100.00236	425	IIIB
TCR12	Chiang Khian, CR	N 19.3652 E 100.00236	425	IIIB	TCR13	Chiang Khian, CR	N 19.36517 E 100.00225	410	IIIB
TCR14	Chiang Khian, CR	N 19.36517 E 100.00225	410	IIIB	TCR15	Chiang Khian, CR	N 19.36517 E 100.00225	410	IIIA
TCR16	Pasang, CR	N 10.11007 E 99.46194	473	IIIB	TCR17	Pasang, CR	N 10.11007 E 99.46194	473	IIIB
TCR18	Pasang, CR	N 10.11007 E 99.46194	473	IIIB	TCR19	Pasang, CR	N 20.11008 E 99.46196	472	IIIA
TCR20	Pasang, CR	N 20.11008 E 99.46196	472	IIIA	TCR21	Pasang, CR	N 20.11008 E 99.46196	472	IIIA
TCR22	Pasang, CR	N 20.11008 E 99.46196	472	IIIA	TCR25	Pasang, CR	N 20.11005 E 99.46221	471	IIIA
TCR26	Pasang, CR	N 20.11005 E 99.46221	471	IIIA	TCR27	Pasang, CR	N 20.10587 E 99.46266	469	IIIA
TCR28	Pasang, CR	N 20.10587 E 99.46266	469	IIIB	TCR29	Pasang, CR	N 20.10587 E 99.46266	469	IIIB
TCR30	Pasang, CR	N 20.10587 E 99.46266	469	IIIB	TCR33	Maekorn, CR	N 19.50299 E 99.39577	481	I
TCR34	Maekorn, CR	N 19.50299 E 99.39577	481	I	TCR35	Maekorn, CR	N 19.50299 E 99.39577	481	I
TCR37	Maekorn, CR	N 19.51419 E 99.41131	497	I	TCR39	Maekorn, Ch	N 19.51419 E 99.41131	497	IIIB
TCR40	Maekorn, CR	N 19.51409 E 99.40115	495	I	TCR41	Maekorn, CR	N 19.51409 E 99.40115	495	IIIB
TCR42	Mae Chedi, CR	N 19.0754 E 99.194	1110	IIIB	TCR43	Mae Chedi, CR	N 19.0754 E 99.194	1110	IIIB
TCR44	Mae Chedi, CR	N 19.08474 E 99.20516	1105	IIIB	TCR45	Mae Chedi, CR	N 19.08474 E 99.20516	1105	IIIC
TCR46	Mae Chedi, CR	N 19.08474 E 99.20516	1105	IIIC	TCR47	Mae Fah Luang, CR	N 20.165067 E 99.44272	660	IIIB
TCR50	Mae Fah Luang, CR	N 20.16077 E 99.44282	649	IIIB	TCR53	Mae Fah Luang, CR	N 20.16077 E 99.44282	649	IIIB
TCR56	Mae Fah Luang, CR	N 20.16076 E 99.44283	639	IIIB	TCR57	Mae Fah Luang, CR	N 20.16002 E 99.44307	670	IIIB
TCR63	Wa wi, CR	N 19.52508 E 99.34497	1780	IIIA	TCR64	Wa wi, CR	N 19.52508 E 99.34497	1780	IIIA
TCR65	Wa wi, CR	N 19.52508 E 99.34497	1780	IIIA	TCR66	Wa wi, CR	N 19.52508 E 99.34497	1780	IIIA
TCR67	Wa wi, CR	N 19.52508 E 99.34497	1780	IIIA	TCR69	Wa wi, CR	N 19.52519 E 99.3453	1696	IIIB
TCR70	Wa wi, CR	N 19.52519 E 99.3453	1696	IIIB	TCR71	Sanklang, CR	N 19.35377 E 99.41371	451	IIIB
TCR73	Huai Chomphu, CR	N 19.52574 E 99.34571	1667	IIIB	TCR74	Huai Chomphu, CR	N 19.52574 E 99.34571	1667	IIIC
TCR75	Huai Chomphu, CR	N 19.53527 E 99.35598	1310	IIIA	TCR76	Huai Chomphu, CR	N 19.53527 E 99.35598	1310	IIIA
TCR77	Huai Chomphu, CR	N 19.53527 E 99.35598	1310	IIIA	TCR78	Huai Chomphu, CR	N 19.53527 E 99.35598	1310	IIIB

Code	Geographical origin	Latitude and longitude	Alt. (m)	Cl.	Code	Geographical origin	Latitude and longitude	Alt. (m)	Cl.
TCR79	Huai Chomphu, CR	N 19.53527 E 99.35598	1310	IIIC	TCR80	Huai Chomphu, CR	N 19.53527 E 99.35598	1310	IIIB
TCR81	Huai Chomphu, CR	N 19.53527 E 99.35598	1310	IIIC	TCR82	Huai Chomphu, CR	N 19.53527 E 99.35598	1310	IIIA
TCR83	Huai Chomphu, CR	N 19.54034 E 99.36521	1286	II	TCR84	Huai Chomphu, CR	N 19.54034 E 99.36521	1286	II
TCR85	Huai Chomphu, CR	N 19.54034 E 99.36521	1286	II	TCR86	Huai Chomphu, CR	N 19.54187 E 99.3801	1183	II
TCR87	Huai Chomphu, CR	N 19.54187 E 99.3801	1183	II	TCR88	Huai Chomphu, CR	N 19.54187 E 99.3801	1183	II
TCM90	Chang Khian, CM	N 18.8377 E 98.89634	1330	IIIB	TCM91	Chang Khian, CM	N 18.85602 E 98.91801	N820	IIIA
TCM92	Chang Khian, CM	N 18.85602 E 98.91801	820	IIIC	TCM93	Chang Khian, CM	N 18.84141 E 98.90244	1340	IIIC
TCM94	Chang Khian, CM	N 18.84215 E 98.90439	1347	IIIC	TCM95	Chang Khian, CM	N 18.84215 E 98.90439	1347	IIIC
TCM96	Chang Khian, CM	N 18.84215 E 98.90439	1347	IIIC	TCM97	Chang Khian, CM	N 18.85217 E 98.91756	905	IIIC
TCM98	Sanpakia, CM	N 19.31414 E 98.83266	1572	IIIC	TCM99	Sanpakia, CM	N 19.31414 E 98.83266	1572	IIIC
TCM100	Sanpakia, CM	N 19.31414 E 98.83266	1572	IIIC	TCM101	Sanpakia, CM	N 19.31414 E 98.83266	1572	IIIC
TCM102	Sanpakia, CM	N 19.31414 E 98.83266	1572	IIIC	TCM103	Sanpakia, CM	N 19.31414 E 98.83266	1572	IIIC
TCM104	Sanpakia, CM	N 19.31414 E 98.83266	1572	IIIC	TCM105	Sanpakia, CM	N 19.31414 E 98.83266	1572	IIIC
TCM106	Sanpakia, CM	N 19.31414 E 98.83266	1572	IIIC	TCM107	Sanpakia, CM	N 19.31414 E 98.83266	1572	IIIC
TCM108	Sanpakia, CM	N 19.31414 E 98.83266	1572	IIIC	TCM109	Sanpakia, CM	N 19.31414 E 98.83266	1572	IIIC
TCM110	Sanpakia, CM	N 19.31414 E 98.83266	1572	IIIC	TCM111	Sanpakia, CM	N 19.31414 E 98.83266	1572	IIIC
TCM112	Pang Hang, CM	N 19.33602 E 98.88393	900	IIIC	TCM113	Pang Hang, CM	N 19.33692 E 98.8845	900	IIIC
TCM114	Pang Hang, CM	N 19.33692 E 98.8845	900	IIIC	TCM115	Pang Hang, CM	N 19.33692 E 98.8845	900	IIIA
TCM116	Pang Hang, CM	N 19.33602 E 98.88393	856	IIIC	TCM117	Pang Hang, CM	N 19.3371 E 98.8845	900	IIIC
TCM118	Pang Hang, CM	N 19.3371 E 98.8845	900	IIIC	TCM119	Pang Hang, CM	N 19.3371 E 98.8845	900	IIIC
TCM120	Pang Hong, CM	N 19.3371 E 98.8845	900	IIIC	TCM121	Pang Hong, CM	N 19.3371 E 98.8845	900	IIIC
TCM122	Pang Hong, CM	N 19.3371 E 98.8845	900	IIIC	TCM123	Pang Hong, CM	N 19.34513 E 98.86799	1084	IIIC
TCM124	Pang Hong, CM	N 19.34513 E 98.86799	1084	IIIC	TCM125	Pang Hong, CM	N 19.34513 E 98.86799	1084	IIIC
TCM126	Pang Hong, CM	N 19.34513 E 98.86799	1084	IIIC	TCM127	Pang Hong, CM	N 19.34513 E 98.86799	1084	IIIC
TCM128a	Pang Hong, CM	N 19.34513 E 98.86799	1084	IIIC	TCM128	Pang Hong, CM	N 19.34513 E 98.86799	1084	IIIC
TCM129	Mae Rim, CM	N 18.87289 E 98.825	780	IIIC	TCM133	Mae Rim, CM	N 18.87051 E 98.82574	794	II
TCM135	Mae Rim, CM	N 18.8654 E98.82632	795	IIIC	TCM139	Mae Rim, CM	N 18.92202 E 98.81563	1047	IIIC
TCM140	Mae Rim, CM	N 18.92202 E 98.81563	1047	IIIC	TCM141	Muser Pak Tang, CM	N 17.57394 E 98.52184	1330	IIIC
TCM142	Muser Pak Tang, CM	N 17.57394 E 98.52184	1330	IIIC	TCM143	Muser Pak Tang, CM	N 17.57394 E 98.52184	1330	IIIC
TCM144	Muser Pak Tang, CM	N 17.57394 E 98.52184	1330	IIIC	TCM145	Muser Pak Tang, CM	N 17.57394 E 98.52184	1330	IIIC

Code	Geographical origin	Latitude and longitude	Alt. (m)	Cl.	Code	Geographical origin	Latitude and longitude	Alt. (m)	Cl.
TCM146	Muser Pak Tang, CM	N 17.57394 E 98.52184	1330	IIIC	TCM147	Muser Pak Tang, CM	N 17.57394 E 98.52184	1330	IIIC
TCM148	Muser Pak Tang, CM	N 17.57394 E 98.52184	1330	IIIC	TCM149	Muser Pak Tang, CM	N 17.57394 E 98.52184	1330	IIIC
TCM150	Muser Pak Tang, CM	N 17.57394 E 98.52184	1330	IIIC	TCM151	Muser Pak Tang, CM	N 17.57394 E 98.52184	1330	IIIC
TCM152	Muser Pak Tang, CM	N 17.57394 E 98.52184	1330	IIIC	TCM153	Muser Pak Tang, CM	N 17.57394 E 98.52184	1330	IIIC
TCM154	Mae Maea, CM	N 19.31414 E 98.83266	1572	IIIC	TCM155	Mae Maea CM	N 19.33692 E 98.8845	900	IIIC
TCM156	Mae Maea, CM	N 19.33692 E 98.8845	900	IIIC	TCM157	Mae Maea, CM	N 19.33602 E 98.88393	856	IIIC
TCM158	Mae Maea, CM	N 19.33602 E 98.88393	856	IIIC	TCM159	Mae Maea, CM	N 19.33602 E 98.88393	856	IIIC
TCM160	Mae Maea, CM	N 19.3371 E 98.8845	900	IIIC	TCM161	Mae Maea, CM	N 19.3371 E 98.8845	900	IIIC
TCM162	Mae Maea, CM	N 19.31965 E 98.89422	756	IIIC	TCM163	Mae Maea, CM	N 19.31975 E 98.8903	733	IIIC
TCM164	Mae Maea, CM	N 19.31975 E 98.8903	733	IIIC	TCM165	Mae Maea, CM	N 19.31975 E 98.8903	733	IIIC
TCM166	Huai Tad, CM	N 19.25928 E 98.91971	992	IIIC	TCM167	Huai Tad, CM	N 19.25928 E 98.91971	992	IIIC
TCM168	Huai Tad, CM	N 19.25928 E 98.91971	992	IIIC	TCM169	Huai Tad, CM	N 19.25928 E 98.91971	992	IIIC
TCM170	Huai Tad, CM	N 19.25928 E 98.91971	992	IIIC	TCM171	Huai Tad, CM	N 19.25928 E 98.91971	992	IIIC
TCM172	Huai Tad, CM	N 19.25928 E 98.91971	992	IIIC	TCM173	Huai Tad, CM	N 19.25928 E 98.91971	992	IIIC
TCM174	Huai Tad, CM	N 19.25928 E 98.91971	992	IIIC	TCM175	Huai Tad, CM	N 19.25928 E 98.91971	992	IIIC
TCM176	Huai Tad, CM	N 19.15908 E 98.90065	389	IIIC	TCM177	Huai Tad, CM	N 19.15908 E 98.90065	389	IIIC
TCM178	Huai Tad, CM	N 19.15908 E 98.90065	389	IIIC	TCM179	Huai Tad, CM	N 19.15908 E 98.90065	389	IIIC
TCM180	Huai Tad, CM	N 19.15908 E 98.90065	389	IIIC	TCM181	Huai Tad, CM	N 19.15908 E 98.90065	389	IIIC
TCM182	Huai Tad, CM	N 19.15908 E 98.90065	389	IIIC	TCM183	Pha Deng, CM	N 19.11708 E 98.72549	900	IIIC
TCM184	Pha Deng, CM	N 19.11708 E 98.72549	900	IIIC	TCM185	Pha Deng, CM	N 19.11708 E 98.72549	900	IIIA
TCM186	Pha Deng, CM	N 19.11708 E 98.72549	900	IIIC	TCM187	Pha Deng, CM	N 19.11708 E 98.72549	900	IIIC
TCM188	Pha Deng, CM	N 19.11708 E 98.72549	900	IIIC	TCM189	Pang Manao, CM	N 19.12659 E 98.70964	847	IIIC
TCM190	Pang Manao, CM	N 19.12659 E 98.70964	847	IIIC	TCM191	Pang Manao, CM	N 19.12659 E 98.70964	847	IIIC
TCM192	Pang Manao, CM	N 19.12659 E 98.70964	847	IIIC	TCM193	Pang Manao, CM	N 19.12659 E 98.70964	847	IIIC
TCM194	Pang Manao, CM	N 19.12659 E 98.70964	847	IIIA	TCM195	Pang Manao, CM	N 19.12659 E 98.70964	847	IIIC
TCM196	Pang Manao, CM	N 19.12659 E 98.70964	847	IIIC	TCM197	Pang Manao, CM	N 19.12659 E 98.70964	847	IIIC
TCM198	Pang Manao, CM	N 19.12659 E 98.70964	847	IIIC	TCM200	Pang Manao, CM	N 19.12659 E 98.70964	847	IIIC
TCM201	Pang Manao, CM	N 19.12659 E 98.70964	847	IIIC	TCM202	Pang Manao, CM	N 19.12659 E 98.70964	847	IIIC
TCM203	Pang Manao, CM	N 19.12659 E 98.70964	847	IIIC	TCM204	Tha Pha, CM	N 19.12638 E 98.75706	662	IIIC
TCM205	Tha Pha, CM	N 19.12638 E 98.75706	662	IIIC	TCM206	Tha Pha, CM	N 19.12638 E 98.75706	662	IIIC

Code	Geographical origin	Latitude and longitude	Alt. (m)	Cl.	Code	Geographical origin	Latitude and longitude	Alt. (m)	Cl.
TCM207	Tha Pha, CM	N 19.12638 E 98.75706	662	IIIC	TCM208	Tha Pha, CM	N 19.11841 E 98.7123	889	IIIC
TCM209	Tha Pha, CM	N 19.11841 E 98.7123	889	IIIC	TCM210	Tha Pha, CM	N 19.11841 E 98.7123	889	IIIC
TCM211	Tha Pha, CM	N 19.11986 E 98.71419	900	IIIC	TCM212	Tha Pha, CM	N 19.12092 E 98.70709	832	IIIC
TCM213	Tha Pha, CM	N 19.12635 E 98.75155	705	IIIC	TCM214	Tha Pha, CM	N 19.12637 E 98.75462	705	IIIC
TCM215	Tha Pha, CM	N 19.12637 E 98.75462	705	IIIC	TCM216	Kiw Tam, CM	N 18.99465 E 99.33821	1111	IIIC
TCM217	Kiw Tam, CM	N 18.99465 E 99.33821	1111	IIIC	TCM218	Kiw Tam, CM	N 18.99465 E 99.33821	1111	IIIC
TCM219	Kiw Tam, CM	N 19.00139 E 99.33125	1157	IIIC	TCM220	Kiw Tam, CM	N 19.00139 E 99.33125	1157	IIIC
TCM221	Kiw Tam, CM	N 19.00139 E 99.33125	1157	IIIC	TCM222	Pang Pong, CM	N 18.97011 E 99.32008	828	IIIC
TCM223	Pang Pong, CM	N 18.97011 E 99.32008	828	IIIC	TCM224	Pang Pong, CM	N 18.97011 E 99.32008	828	IIIC
TCM225	Pang Pong, CM	N 18.97011 E 99.32008	828	IIIC	TCM226	Kam Phang Hin, CM	N 18.9489 E 99.35128	1083	IIIC
TCM227	Kam Phang Hin, CM	N 18.9489 E 99.35128	1083	IIIB	TCM228	Kam Phang Hin, CM	N 18.9489 E 99.35128	1083	IIIC
TCM229	Kam Phang Hin, CM	N 18.9489 E 99.35128	1083	IIIC	TCM230	Kam Phang Hin, CM	N 18.9489 E 99.35128	1083	IIIC
TCM231	Kam Phang Hin, CM	N 18.9489 E 99.35128	1083	IIIC	TCM232	Kam Phang Hin, CM	N 18.9489 E 99.35128	1083	IIIC
TCM233	Kam Phang Hin, CM	N 18.9489 E 99.35128	1083	I	TCM234	Kam Phang Hin, CM	N 18.9489 E 99.35128	1083	IIIC
TCM235	Mae Ton, CM	N 18.93543 E 99.37676	1436	IIIC	TCM236	Mae Ton, CM	N 18.93543 E 99.37676	1436	IIIC
TCM237	Mae Ton, CM	N 18.93543 E 99.37676	1436	IIIC	TCM238	Pang Hi, CM	N 18.92282 E 99.33241	950	IIIC
TCM239	Ban Dong, CM	N 18.92399 E 99.35216	1157	IIIC	TCM240	Ban Dong, CM	N 18.92399 E 99.35216	1157	IIIC
TCM241	Ban Dong, CM	N 18.92399 E 99.35216	1157	IIIC	TN242	Reuang, Nan	N 18.47007 E 100.39187	282	IIIA
TN243	Reuang, Nan	N 18.47336 E 100.38005	351	IIIA	TN244	Reuang, Nan	N 18.47336 E 100.38005	351	IIIA
TN245	Reuang, Nan	N 18.47336 E 100.38005	351	IIIA	TN246	Reuang, Nan	N 18.47324 E 100.38000	358	IIIA
TN247	Reuang, Nan	N 18.47314 E 100.38074	355	IIIA	TN248	Reuang, Nan	N 18.47314 E 100.38074	355	IIIA
TN249	Reuang, Nan	N 18.47307 E 100.3827	384	IIIA	TN250	Reuang, Nan	N 18.47307 E 100.3827	384	IIIA
TN251	Reuang, Nan	N 18.47307 E 100.3827	384	IIIA	TN252	Sakad A, Nan	N 19.16021 E 101.00441	1033	IIIA
TN253	Sakad A, Nan	N 19.16003 E 101.00459	1059	IIIA	TN254	Sakad A, Nan	N 19.15588 E 101.00374	1053	IIIA
TN255	Sakad A, Nan	N 19.15588 E 101.00374	1053	IIIA	TN256	Sakad A, Nan	N 19.15588 E 101.00374	1053	IIIA
TN257	Sakad A, Nan	N 19.15588 E 101.00374	1053	IIIA	TN258	Sakad A, Nan	N 19.15588 E 101.00374	1053	IIIA
TN259	Sakad A, Nan	N 19.15588 E 101.00374	1053	IIIA	TN260	Sakad A, Nan	N 19.15588 E 101.00374	1053	IIIA
TN261	Sakad A, Nan	N 19.15588 E 101.00374	1053	IIIA	TN262	Sakad A, Nan	N 19.16056 E 101.00587	1090	IIIA
TN263	Sakad A, Nan	N 19.16056 E 101.00587	1090	IIIA	TN264	Sakad A, Nan	N 19.16056 E 101.00587	1090	IIIA
TN265	Sakad A, Nan	N 19.16092 E 101.01003	1083	IIIA	TN266	Sakad A, Nan	N 19.16092 E 101.01003	1083	IIIA

Code	Geographical origin	Latitude and longitude	Alt. (m)	Cl.	Code	Geographical origin	Latitude and longitude	Alt. (m)	Cl.
TN267	Sakad A, Nan	N 19.16092 E 101.01003	1083	IIIA	TN268	Sakad B, Nan	N 19.16036 E 101.01097	1148	IIIA
TN269	Sakad B, Nan	N 19.16036 E 101.01097	1148	IIIA	TN270	Sakad B, Nan	N 19.16036 E 101.01097	1148	IIIA
TN271	Sakad B, Nan	N 19.16045 E 101.01059	1146	IIIA	TN272	Sakad B, Nan	N 19.16045 E 101.01059	1146	IIIA
TN273	Sakad B, Nan	N 19.16049 E 101.0104	1141	IIIA	TN274	Sakad B, Nan	N 19.6038 E 101.01017	1128	IIIA
TN275	Sakad B, Nan	N 19.16056 E 101.00587	1090	IIIA	TN277	Sakad B, Nan	N 19.16056 E 101.00587	1090	IIIA
TN278	Sakad B, Nan	N 19.16092 E 101.01003	1083	IIIA	TN279	Sakad B, Nan	N 19.16092 E 101.01003	1083	IIIA
TN280	Sakad B, Nan	N 19.16092 E 101.01003	1083	IIIA	TP281	Pa Daeng, Phrae	N 18.02288 E 101.19042	391	IIIA
TP282	Pa Daeng, Phrae	N 18.02288 E 101.19042	391	IIIA	TP283	Pa Daeng, Phrae	N 18.02288 E 101.19042	391	IIIA
TP284	Pa Daeng, Phrae	N 18.02288 E 100.19046	401	IIIA	TP285	Pa Daeng, Phrae	N 18.02287 E 100.19028	440	IIIA
TP286	Pa Daeng, Prae	N 18.02287 E 100.19028	440	IIIA	TP289	Pa Daeng, Phrae	N 18.02282 E 100.19027	433	IIIA
TP290	Pa Daeng, Phrae	N 18.02282 E 101.19027	433	IIIA	TP291	Pa Daeng, Phrae	N 18.02289 E 100.19026	430	IIIA
TP293	Pa Daeng, Phrae	N 18.02293 E 100.19033	406	IIIA	TMS295	Mai Hung, MS	N 19.62125 E 98.20842	1105	IIIA
TMS296	Mai Hung, MS	N 19.62125 E 98.20842	1105	IIIA	TMS297	Mai Hung, MS	N 19.62125 E 98.20842	1105	IIIA
TMS298	Mai Hung, MS	N 19.62125 E 98.20842	1105	IIIA	TMS300	Mai Hung, MS	N 19.5928 E 98.21405	788	IIIA
TMS301	Mai Hung, MS	N 19.5928 E 98.21405	788	IIIA	TMS302	Mai Hung, MS	N 19.62125 E 98.20842	1105	IIIA
TMS303	Mai Hung, MS	N 19.62125 E 98.20842	1105	IIIA	TMS304	Mai Hung, MS	N 19.62125 E 98.20842	1105	IIIA
TMS306	Mae La Na, MS	N 19.59016 E 98.21456	777	IIIA	TMS308	Mae La Na, MS	N 19.59016 E 98.21456	777	IIIA
TLP309	Chae Son, LP	N 18.49574 E 99.2315	998	IIIA	TLP310	Chae Son, LP	N 18.50067 E 99.23322	1039	IIIA
TLP311	Chae Son, LP	N 18.50078 E 99.2335	1099	IIIA	TLP312	Chae Son, LP	N 18.50067 E 99.23381	1081	IIIA
TLP313	Ban Rong, LP	N 19.02328 E 99.4693	833	IIIA	TLP314	Ban Rong, LP	N 19.02306 E 99.46481	870	IIIA
TLP315	Ban Rong, LP	N 19.0229 E 99.46486	866	IIIA	TLP316	Ban Rong, LP	N 19.02283 E 99.46484	865	IIIA
TLP317	Ban Rong, LP	N 19.02269 E 99.46473	861	IIIA	TLP318	Ban Rong, LP	N 19.02582 E 99.46272	843	IIIA
TLP319	Ban Rong, LP	N 19.02582 E 99.46272	843	IIIA					

Note: Alt. indicates Altitude; Cl., cluster; CR, Chiang Rai province; CM, Chaing Mai province; MS, Mae Hong Son province; LP, Lampang province.
